# T45. A COMPARISON OF SCHIZOPHRENIA RELAPSE RATES OF 3 PALIPERIDONE FORMULATIONS, ONCE-DAILY, ONCE-MONTHLY AND ONCE EVERY-3-MONTH: POST-HOC ANALYSIS FROM 3 RANDOMIZED CONTROLLED TRIALS

**DOI:** 10.1093/schbul/sby016.321

**Published:** 2018-04-01

**Authors:** Maju Mathews, Srihari Gopal, Arun Singh, Isaac Nuamah, Anne Marie Quinn, Katalin Pungor, Wilson Tan, Bernardo Soares, Edward Kim

**Affiliations:** 1 Janssen Inc; 2 Janssen-Cilag

## Abstract

**Background:**

Poor adherence to antipsychotic treatment in patients with schizophrenia can result in recurrent relapses, worsening disease, functional impairment and reduction in treatment responsiveness. Long-acting antipsychotic formulations can maintain therapeutic plasma levels for longer durations, reducing dosing frequency and delaying time to relapse compared to oral formulations. Consequently, relatively lower rates of relapse can be expected in patients on long-acting injectables (LAIs) who have discontinued treatment versus those discontinuing oral medications of the same antipsychotic. However, there is no available evidence to support this association. In this post hoc analysis, the percentage of patients who relapsed and the time to relapse for three different formulations of the same molecule (oral paliperidone extended release [ER]; paliperidone palmitate once monthly [PP1M] LAI, and paliperidone palmitate three monthly [PP3M] LAI) were evaluated in adults with schizophrenia, comparing the active and placebo arms.

**Methods:**

Data from three similarly designed, randomized, double-blind, placebo-controlled relapse prevention studies in adult patients with schizophrenia (DSM-IV-TR criteria) with similar inclusion/exclusion and relapse criteria were analyzed. Patients stabilized during an open-label stabilization phase with either paliperidone ER, PP1M or PP3M were then randomized to receive either placebo (analogous to non-adherent patients in the real-world) or the same active treatment used during stabilization phase (analogous to adherent patients). The primary outcome in each study was the time to relapse after entering the randomization phase, estimated using Kaplan-Meier method. In this report, the percentage of patients who relapsed as well as time to relapse in the three studies were indirectly compared.

**Results:**

In total 922 patients were included in this analysis, 473 continued to receive the same active treatment and 449 patients were randomized to receive placebo. The percentage of patients who relapsed was lowest with PP3M as compared with PP1M and paliperidone ER in both the active treatment group (PP3M, 9% < PP1M, 18% < paliperidone ER, 22%) and placebo group (PP3M, 29% < PP1M, 48% < paliperidone ER, 52%) patients. The post discontinuation median time to relapse (95% confidence interval) in placebo group was highest with PP3M, 395 days (274 days to not reached) > PP1M, 172 days (134 to 222 days) > paliperidone ER, 58 days (42 to 114 days) but was not estimable in the paliperidone group.

**Discussion:**

Treatment with longer acting formulations of paliperidone are associated with lower percentage of relapse and longer time to relapse in patients with schizophrenia. Lower percentage of patients with relapse observed with LAI therapy (PP1M and PP3M) could presumably be due to ensured therapeutic plasma levels. The lower percentage of relapse observed with PP3M treatment as compared with PP1M and oral paliperidone ER treatment in the placebo group could be advantageous to non-adherent patients, as this mimics the real-world scenario where patients discontinue their antipsychotics suddenly. These findings are of relevance in schizophrenia patients as fewer and delayed relapses over the course of a lifetime of schizophrenia may provide higher protection against grey matter damage and help preserve functioning.

